# Dental Resin Cements—The Influence of Water Sorption on Contraction Stress Changes and Hydroscopic Expansion

**DOI:** 10.3390/ma11060973

**Published:** 2018-06-08

**Authors:** Grzegorz Sokolowski, Agata Szczesio, Kinga Bociong, Karolina Kaluzinska, Barbara Lapinska, Jerzy Sokolowski, Monika Domarecka, Monika Lukomska-Szymanska

**Affiliations:** 1 Department of Prosthetic Dentistry, Medical University of Lodz, 251 Pomorska St., 92-213 Lodz, Poland; grzegorz.sokolowski@umed.lodz.pl; 2 University Laboratory of Materials Research, Medical University of Lodz, 251 Pomorska St., 92-213 Lodz, Poland; agata.szczesio@umed.lodz.pl (A.S.); kinga.bociong@umed.lodz.pl (K.B.); karolina.kaluzinska@umed.lodz.pl (K.K.); 3 Department of General Dentistry, Medical University of Lodz, 251 Pomorska St., 92-213 Lodz, Poland; barbara.lapinska@umed.lodz.pl (B.L.); jerzy.sokolowski@umed.lodz.pl (J.S.); monika.domarecka@umed.lodz.pl (M.D.)

**Keywords:** resin cements, shrinkage stress, water sorption, hydroscopic expansion, photoelastic investigation

## Abstract

Resin matrix dental materials undergo contraction and expansion changes due to polymerization and water absorption. Both phenomena deform resin-dentin bonding and influence the stress state in restored tooth structure in two opposite directions. The study tested three composite resin cements (Cement-It, NX3, Variolink Esthetic DC), three adhesive resin cements (Estecem, Multilink Automix, Panavia 2.0), and seven self-adhesive resin cements (Breeze, Calibra Universal, MaxCem Elite Chroma, Panavia SA Cement Plus, RelyX U200, SmartCem 2, and SpeedCEM Plus). The stress generated at the restoration-tooth interface during water immersion was evaluated. The shrinkage stress was measured immediately after curing and after 0.5 h, 24 h, 72 h, 96 h, 168 h, 240 h, 336 h, 504 h, 672 h, and 1344 h by means of photoelastic study. Water sorption and solubility were also studied. All tested materials during polymerization generated shrinkage stress ranging from 4.8 MPa up to 15.1 MPa. The decrease in shrinkage strain (not less than 57%) was observed after water storage (56 days). Self-adhesive cements, i.e., MaxCem Elite Chroma, SpeedCem Plus, Panavia SA Plus, and Breeze exhibited high values of water expansion stress (from 0 up to almost 7 MPa). Among other tested materials only composite resin cement Cement It and adhesive resin cement Panavia 2.0 showed water expansion stress (1.6 and 4.8, respectively). The changes in stress value (decrease in contraction stress or built up of hydroscopic expansion) in time were material-dependent.

## 1. Introduction

Resin composite cements have been widely used with ceramic, resin, or metal alloy-based prosthodontic restorations [1]. The cementation technique used in adhesive dentistry is one of the major factors, which exerts influence on the clinical success of indirect restorative procedures. Cement is used to bond tooth and restoration simultaneously creating a barrier against microbial leakage [2]. The universal cement that can be applied in all indirect restorative procedures has not been introduced into the market yet. Therefore, clinicians should understand the influence of applied material properties and preparation design on the clinical performance of the restoration [3].

**Figure 1:**
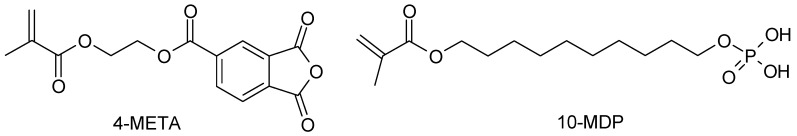
Monomers used as bonding agents in resin cements.

The composition of resin composite cements is almost the same as of resin composites [4]. Resin cements mainly consist of various methacrylate resins and inorganic fillers which are often coated with organic silanes to provide adhesion between the filler and the matrix. These materials often include bonding agents to promote the adhesion between resin cement and tooth structure. Main monomers are, i.e., hydroxyethyl methacrylate (HEMA), 4-methacryloyloxyethy trimellitate anhydride (4-MET), carboxylic acid, and organophospate 10-methacryloxydecyl dihydrogen phosphate (10-MDP) (Figure 1). The acidic group bonds calcium ions in the tooth structure [5,6].

Resin composite cements are used in combination with adhesive systems. This procedure aims at creating micro-mechanical retention to both enamel and dentin. The material may also form a strong adhesion to an adequately-treated surface of the composite, ceramic, and metallic restorations [7]. Taking into account the surface preparation before the cementation process, resin cements can be divided into: (1) composite resin cement (used with total-etch adhesive systems); (2) adhesive resin cement (used with separate self-etching adhesive systems); and (3) self-adhesive resin cement (containing a self-adhesive system) [1].

The application of resin matrix-based cements is time-consuming and susceptible to manipulation errors [8]. The self-adhesive resin cements are proposed to simplify the restoration procedure. These materials bond dentin in one step without any surface conditioning or pre-treatment (priming) [9,10].

All currently available resin-based materials exhibit polymerization shrinkage. Moreover, resin cements are generally applied as a thin layer, particularly when used to lute posts, inlays, and crowns. In the aforementioned clinical cases, the cavity design has a high C-factor (high number of bonded surfaces and a low number of un-bonded surfaces) [11]. Additionally, low-viscosity composites exhibit a relatively high shrinkage amounting up to 6% (comparable to resin cements) [12,13]. These factors may generate sufficient stress resulting in debonding of the luting material, thereby increasing microleakage [14]. Nevertheless, there is little data on the stress generated by these materials. The sorption characteristic of resin-based dental cements has been extensively evaluated [15,16,17]. However, the analysis of the influence of water sorption on the change in contraction stress is inadequate. The purpose of this study was to evaluate the development of the stress state, i.e., the contraction stress generated during photopolymerization and hydroscopic expansion within different types of resin cements which undergo water ageing by means of photoelastic analysis.

## 2. Materials and Methods

The composition of investigated resin cements and bonding systems is presented in Table 1 and Table 2.

### 2.1. Absorbency Dynamic Study

Absorbency dynamic was determined by means of procedure as described by Bociong et al. [18]. The samples were prepared according to ISO 4049 [19]. Curing time was consistent with the manufacturer’s instructions (Table 2).

In order to characterize absorbency dynamic, the cylindrical samples with dimensions of 15 mm in diameter and of 1 mm in width were prepared. The tested materials were applied in one layer and cured with LED light lamp (Mini L.E.D., Acteon, Mérignac Cedex, France) in nine zones partially overlapping each other with direct contact of optical fiber with the material surface. Exposure time was applied according to the manufacturer’s instructions (Table 1).

Five samples were prepared for each tested cement. The samples’ weight was determined (RADWAG AS 160/C/2, Poland) immediately after preparation and then for 30 consecutive days, and after 1344 h (56 days) and 2016 h (84 days). The absorbency was calculated according to the Equation (1) [20]:

(1) $$A=\frac{m_{i}-m_{0}}{m_{0}}\cdot 100\%$$

where A is the absorbency of water, m_0_ is the mass of the sample in dry condition, and m_i_ is the mass of the sample after storage in water for a specified (i) period of time.

### 2.2. Water Sorption and Solubility

Water sorption and solubility were investigated according to ISO 4049 [19]. The detailed procedure of tests has been described extensively in the previously published literature [18,21]. Curing time was consistent with the manufacturer’s instructions (Table 1).

Water sorption and solubility were calculated according to following equations:

(2) $$W_{sp}=\frac{m_{2}-m_{3}}{V}$$

(3) $$W_{sl}=\frac{m_{1}-m_{3}}{V}$$

where: *W_sp_* is the water sorption, *W_sl_* is the water solubility, *m*_1_ is the initial constant mass (μg), *m*_2_ is the mass after seven days of water immersion (μg), *m*_3_ is the final constant mass (μg), *V* is the specimen volume (mm^3^).

### 2.3. Photoelastic Study

Photoelastic analysis allows for quantitative measurement and visualization of stress concentration that develops during photopolymerization or water sorption of resin composites [22,23]. The modified method enables analysis of the relationship between water sorption and the change of stress state (contraction or expansion) of resin materials. This test was described extensively in our previous articles [18,24]. Photoelastically-sensitive plates of epoxy resin (Epidian 53, Organika-Sarzyna SA, Nowa Sarzyna, Poland) were used in this study. Calibrated orifices of 3 mm in diameter and of 4 mm in depth were prepared in resin plates in order to mimic an average tooth cavity. The generated strains in the plates were visualized in circular transmission polariscope FL200 (Gunt, Barsbüttel, Germany) and photoelastic strain calculations were based on the Timoshenko equation [25].

## 3. Results

### 3.1. Absorbency Dynamic Study

Water absorbency and contraction stress mean values were presented in Figure 2, Figure 3, Figure 4, Figure 5, Figure 6, Figure 7, Figure 8, Figure 9, Figure 10, Figure 11, Figure 12, Figure 13 and Figure 14. The water immersion resulted in an increase in weight of all tested materials. The water sorption (wt%) increased for Breeze up to three times. The lowest value of absorbency after 2016 h (84 days) was observed for Variolink Esthetic DC.

### 3.2. Water Sorption and Solubility

Mean values of water sorption and solubility were presented in Table 3. Maxcem Elite Chroma and Breeze exhibited the highest, while Estecem showed the lowest values of water sorption.

### 3.3. Photoelastic Study

All materials exhibited shrinkage and the associated contraction stress during hardening process. The significant reduction in contraction stress was observed due to hygroscopic expansion of cements (Figure 2, Figure 3, Figure 4, Figure 5, Figure 6, Figure 7, Figure 8, Figure 9, Figure 10, Figure 11, Figure 12, Figure 13, Figure 14, Figure 15 and Figure 16). Water ageing of six cements resulted in additional stress characterized by the opposite direction of forces. The investigated materials exhibited various contraction stress values.

The lowest contraction stress from tested materials exhibited Panavia SA Plus. The contraction stress decreased from 4.8 up to 0.0 MPa after 504 h (21 days) of water conditioning (Figure 11). Further water ageing resulted in additional stress: after 2016 h (84 days) stress level increased up to −1.6 MPa (Figure 11).

The highest contraction stress was observed for SmartCem 2 amounting up to 15.1 MPa. The contraction stress of SmartCem 2 after 2016 h (84 days) of water storage reduced up to 1.6 MPa (Figure 13).

## 4. Discussion

High configuration factor (C-factor) and the low viscosity of resin cements may generate relatively high contraction stress. This stress may cause debonding of the luting material, thereby increasing microleakage [14]. Our previous study (using an epoxy resin plate) [18,24], demonstrated that the photoelastic method can be used to evaluate the effect of water sorption on stress reduction at the tooth-restoration interface. This method shows that the contraction stress of dental resins may be partially relieved by the water uptake [18]. However, the in-depth analysis of the shrinkage stress values in various resin cement materials after water ageing is also highly demanded.

The overall results showed the development of the initial stress in the compressive direction during photopolymerization. The composition of resin cements affected the sorption and solubility processes which, in turn, exerted influence on the hygroscopic expansion and plasticization. Thus, the compensatory effect was composition-dependent [26]. This study confirmed the lower water sorption for composite resin cements as compared with self-adhesive resin cements that underwent water ageing. The presence of hydroxyl, carboxyl, and phosphate groups in monomers made them more hydrophilic and, supposedly, more prone to water sorption [27].

Variolink Esthetic DC and NX3 do not contain adhesive monomers. In the present study they exhibited sorption similar to composite materials [18]. Variolink Esthetic DC showed a high solubility value and the lowest decrease in contraction stress (of about 70%). The water immersion of resin materials might result in dissolving and leaching of some components (unreacted monomers or fillers) out of the material [28]. Variolink Esthetic DC contains a modified polymer matrix and nanofiller. The lowest contraction stress might result from a small hydrolysis and plasticization effect of the modified resin matrix.

Cement It is a composite resin cement which, in comparison with NX3 and Variolink Esthetic DC, showed higher values of water sorption and its total value of stress changes was 12.5 MPa. This could be explained by the composition of the polymer matrix containing bis-GMA and HDDMA. These monomers showed comparable characteristics: high water sorption values [29,30], similar polymer networks, and susceptibility to hydrolysis [31].

**Figure 2:**
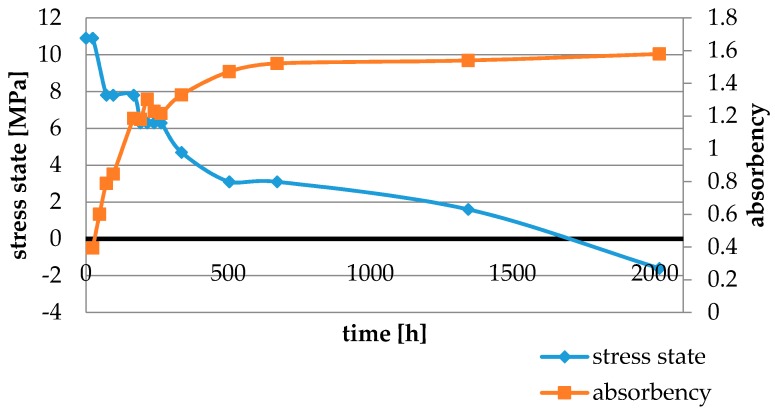
The influence of water sorption (2016 h water ageing) on the absorbency and contraction stress generated during the photopolymerization of Cement It.

**Figure 3:**
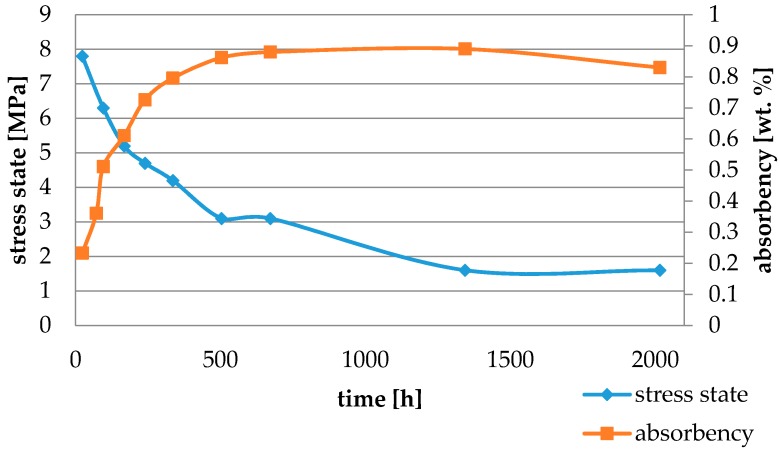
The influence of water sorption (2016 h water ageing) on the absorbency and contraction stress generated during the photopolymerization of NX3.

**Figure 4:**
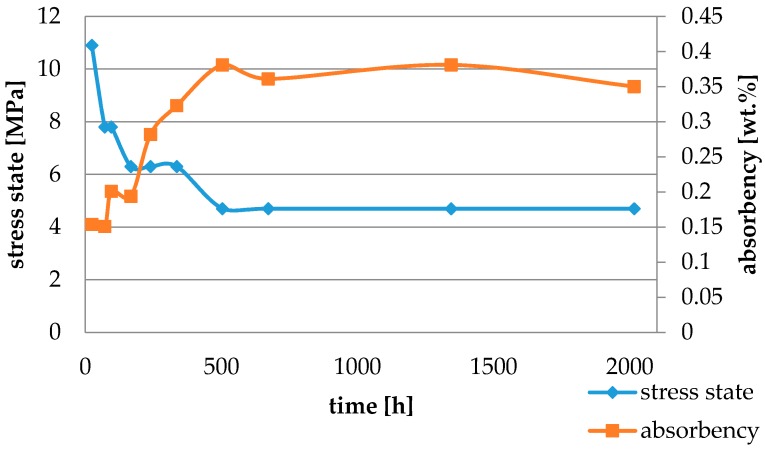
The influence of water sorption (2016 h water ageing) on the absorbency and contraction stress generated during the photopolymerization of Variolink Esthetic DC.

**Figure 5:**
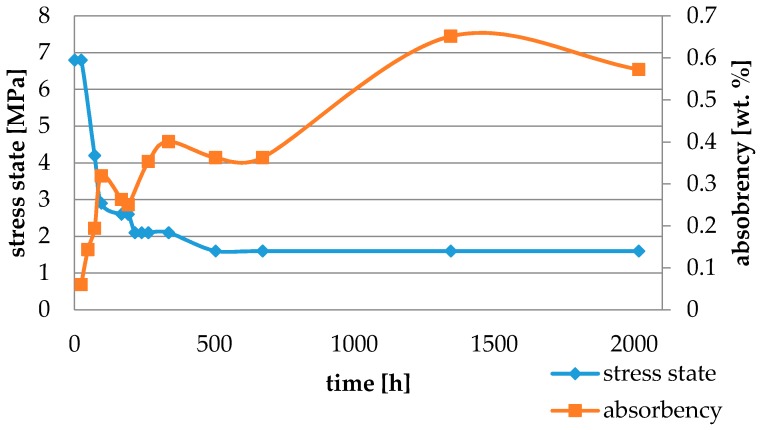
The influence of water sorption (2016 h water ageing) on the absorbency and contraction stress generated during the photopolymerization of Estecem.

**Figure 6:**
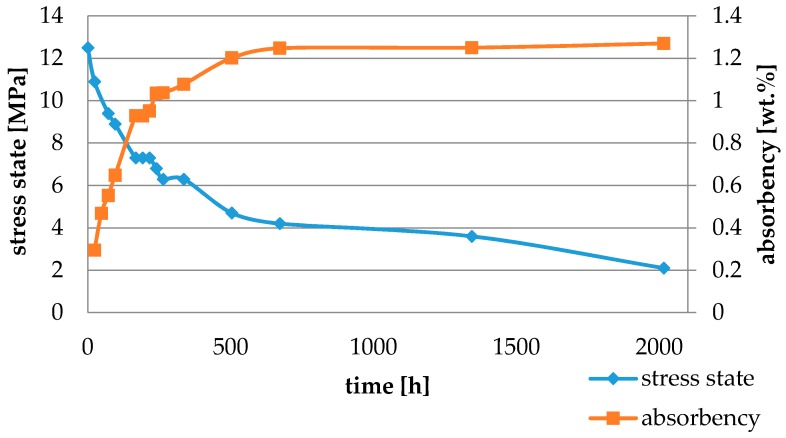
The influence of water sorption (2016 h water ageing) on the absorbency and contraction stress generated during the photopolymerization of Multilink Automix.

**Figure 7:**
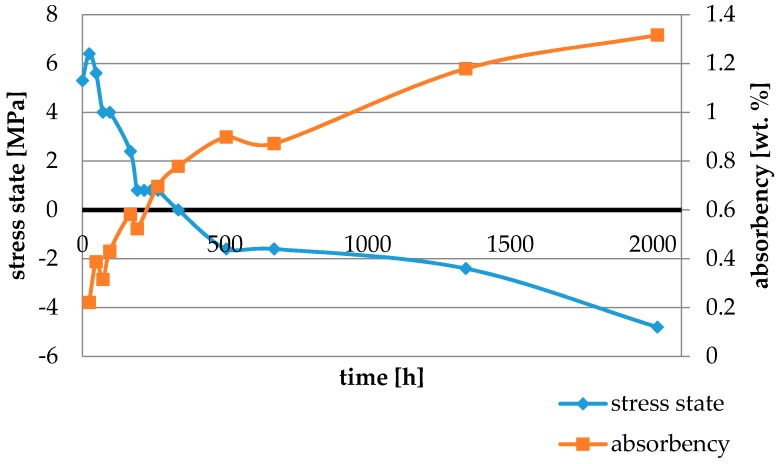
The influence of water sorption (2016 h water ageing) on the absorbency and contraction stress generated during the photopolymerization of Panavia 2.0.

**Figure 8:**
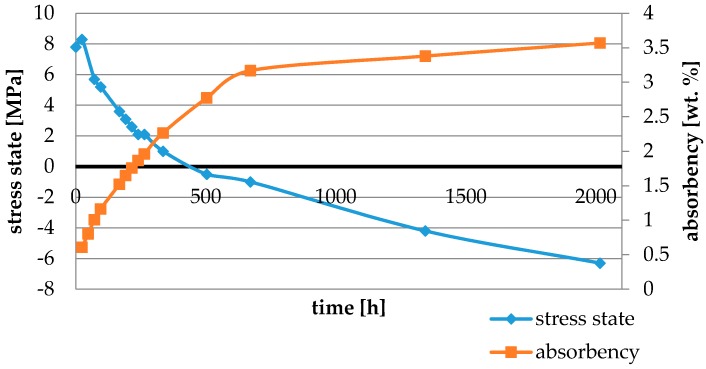
The influence of water sorption (2016 h water ageing) on the absorbency and contraction stress generated during the photopolymerization of Breeze.

**Figure 9:**
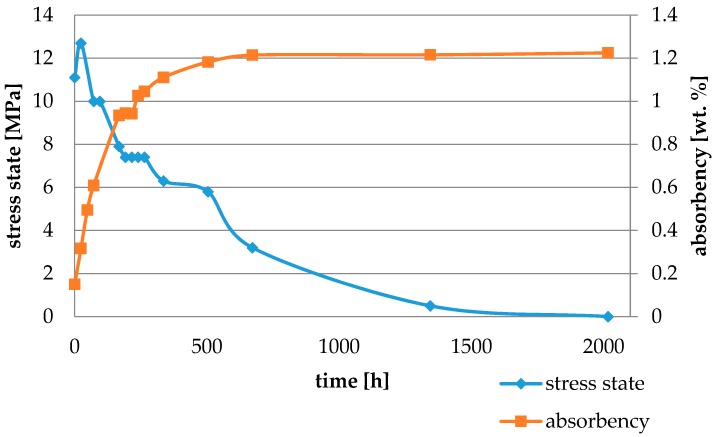
The influence of water sorption (2016 h water ageing) on the absorbency and contraction stress generated during the photopolymerization of Calibra Universal.

**Figure 10:**
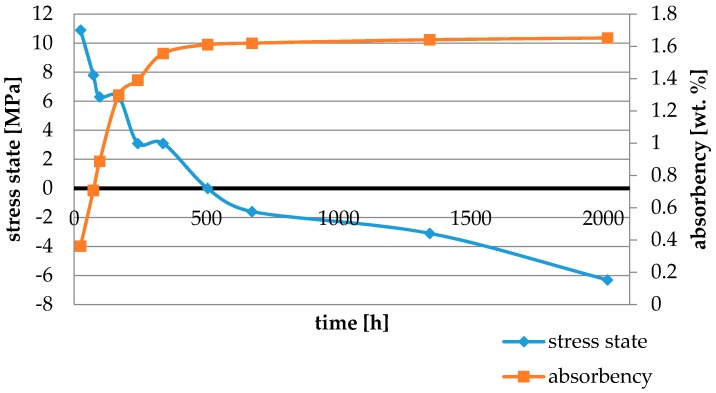
The influence of water sorption (2016 h water ageing) on the absorbency and contraction stress generated during the photopolymerization of Maxcem Elite Chroma.

**Figure 11:**
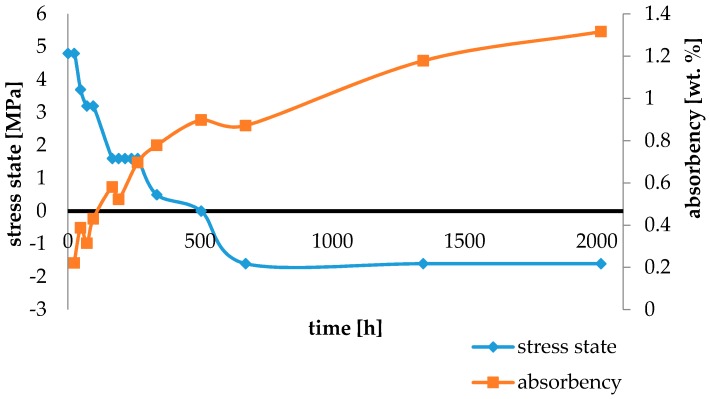
The influence of water sorption (2016 h water ageing) on the absorbency and contraction stress generated during the photopolymerization of Panavia SA Plus.

**Figure 12:**
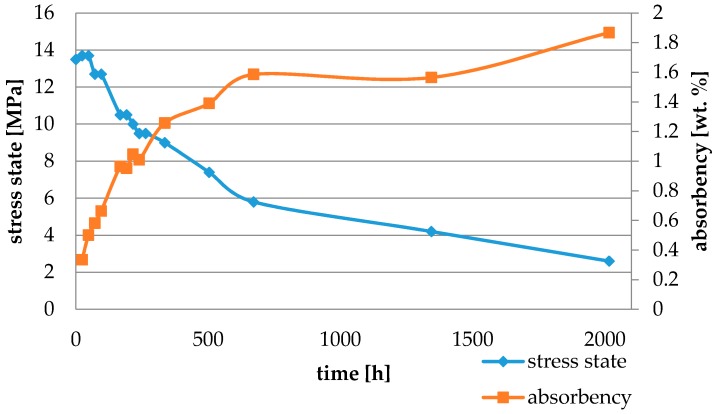
The influence of water sorption (2016 h water ageing) on the absorbency and contraction stress generated during the photopolymerization of Rely U200.

**Figure 13:**
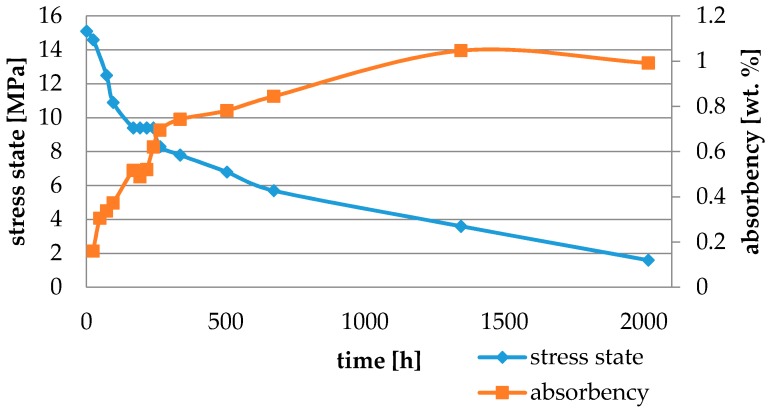
The influence of water sorption (2016 h water ageing) on the absorbency and contraction stress generated during the photopolymerization of SmartCem 2.

**Figure 14:**
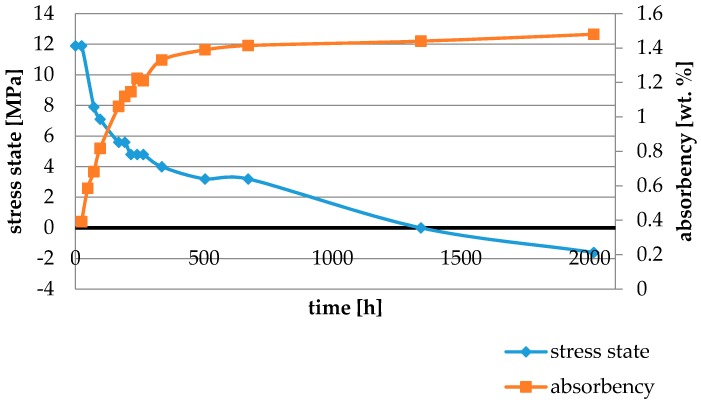
The influence of water sorption (2016 h water ageing) on the absorbency and contraction stress generated during the photopolymerization of SpeedCEM Plus.

**Figure 15:**
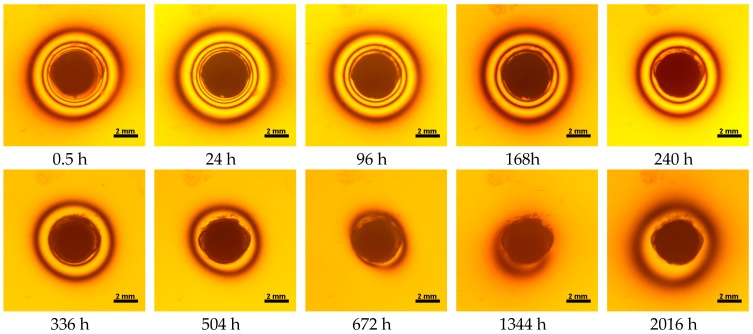
Isochromes in an epoxy plate around Maxcem Elite Chroma restoration before and after water storage; 0.5–2016 h.

**Figure 16:**
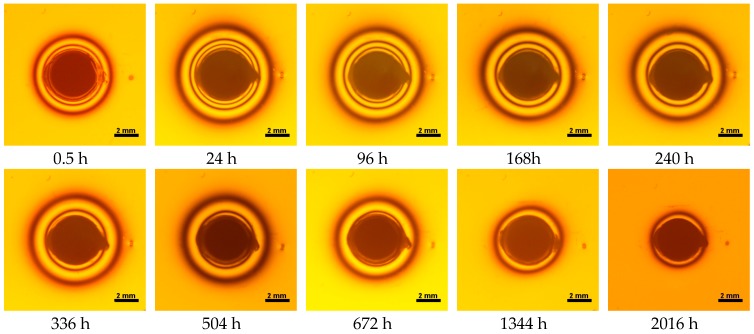
Isochromes in an epoxy plate around NX3 restoration before and after water storage; 0.5–2016 h.

The four tested materials did not meet the requirements of ISO 4049, as they showed sorption values above 40 μg/mm^3^ (Breeze and MaxCem Elite Chroma) or the solubility value above 7.5 μg/mm^3^ (Variolink Esthetic DC and Panavia 2.0). The differences in water absorption of the polymer network depending on monomer type were reported. The highest water sorption was observed for MaxCem Elite Chroma (Figure 15). This material consists of HEMA and GDM that have one of the highest hydrophilicities among dental resins. HEMA was shown to induce water sorption, leading to the expansion of the polymer matrix [32]. Resin-modified glass-ionomer cements (RMGICs) absorbed more water due to hydroxyethyl methacrylate content, present in the hydrogel form in the polymerized matrices [33]. HEMA might be present either as a separate component or a grafted component into the structure of the polyacrylic acid backbone. The polymerized matrices of these materials were very hydrophilic and might include an interpenetrating network of poly(HEMA), copolymers of grafted HEMA, and polyacid salts that were more prone to water uptake [34]. Park et al. [35] showed that GDM exhibited the highest water sorption in comparison to bis-GMA, HEMA, EGDM, DEGDM, TEGDMA, GDM, and GTM. In the present study, the decrease in contraction stress after 2016 h (84 days) of water immersion varied significantly between tested materials. Figure 2, Figure 3, Figure 4, Figure 5, Figure 6, Figure 7, Figure 8, Figure 9, Figure 10, Figure 11, Figure 12, Figure 13, Figure 14, Figure 15 and Figure 16 showed that the expansion dynamics also differed substantially. All studied materials exhibited contraction stress relief during water immersion. The value of stress decreased up to 0 MPa in different times depending on the material and its composition.

To sum up, the phenomenon of hydroscopic expansion after compensation of contraction stress should be emphasized more and evaluated. Such over-compensation could lead to internal expansion stress [36]. Hygroscopic stress could result in micro-cracks or even cusp fractures in the restored tooth [37], poorer mechanical properties [38,39], hydrolytic degradation of bonds particularly at the resin–filler interface [40], polymer plasticization leading to hardness reduction and glass transition temperature [40], and impaired wear resistance [41]. Excessive water sorption is not desired as it causes an outward movement of residual monomers and ions due to material solubility. Furthermore, water sorption might generate peeling stress in bonded layers of polymers that may cause serious clinical consequences [42], which may occur especially when prosthetic restorations are adhesively cemented [42].

The present study demonstrated that self-adhesive cements, i.e., Maxcem Elite Chroma, Speed Cem Plus, Cement It, Panavia SA Plus, Breeze, and Panavia 2.0 exhibited high stress values due to water expansion (from 0 up to almost 7 MPa). Water expansion stress of Maxcem Elite Chroma and Breeze amounted to up to ~6–7 MPa which could be associated with their composition, particularly with acidic monomer 4-MET in Breeze and HEMA, and GDM and tetramethylbutyl hydroperoxide in Maxcem Elite Chroma. The monomers, mentioned above, were responsible for water uptake and stress built-up associated with hydroscopic expansion [35]. According to the literature such high stress values were not desirable. Huang et al. [33] found that a giomer material exhibited extensive hygroscopic expansion (due to osmotic effect) enabling enclosing glass cylinders to crack after two weeks of immersion in water. Cusp fracture in endodontically-treated teeth was attributed to hygroscopic expansion of a temporary filling material [33], while cracks in all-ceramic crowns were associated with hygroscopic expansion of compomer and resin-modified glassionomer materials used as core build-up and/or luting cements [33]. Three-year clinical performance study also suggested hygroscopic expansion as a possible cause of cusp fracture in 19% of teeth restored with an ion-releasing composite [43].

Thus, the positive influence of water sorption on contraction stress relief in the case of luting cements, particularly self-adhesive materials, should be considered carefully. Shrinkage occurred within seconds, but water sorption took days and weeks. The rate of hygroscopic shear stress relief depended on the resin volume and its accessibility to water [11]. The contraction stress relief rates observed in luting resin cements could be much lower than in composite resin restorative materials. The composite restorations usually have a relatively large surface exposed to water in comparison to the overall surface of the luting material. As far as luting cements are concerned, the surface exposed to oral fluids is extremely small, while the pathway is extremely long. The consequences (slower compensation of contraction stress) might be less severe if water sorption is also possible from the dentin (dentin exposed to oral environment) [26].

The precise effect of water absorption depends on many factors including not only the material characteristics, the rate and amount of water absorbed, but also the mechanism of absorption [44]. Absorption leads to dimensional changes and has potentially important clinical implications. The positive effect of water absorption on composite restorative materials can be described as the mechanism for the compensation of polymerization shrinkage and the relaxation of stress. In clinical conditions, water absorption may help in the closure of contraction gaps around composite filling materials. It is worth emphasizing that the absorption can, in some cases, result in significant hydroscopic expansion and, thus, be damaging to the resin material and bonded tooth structure.

## 5. Conclusions

Among all studied resin cements, self-adhesive cements exhibited the highest water sorption due to acid monomer content, which affected the formation of hydroscopic expansion stress. The presence of this type of stress might pose a threat to prosthetic restorations. Therefore, there is still a need for research that would precisely illustrate the generated stress in clinical conditions.

Tested resin cements generated differentiated contraction stress during photopolymerization. The dynamic of hydroscopic compensation (resulting from water sorption) or over-compensation of the contraction stress is also material characteristic-dependent.

## Figures and Tables

**Table 1:** The composition of resin cements.

Material	Type	Composition	Curing Time (s)	Manufacturer
Cement-It	Composite resin cement	bis-GMA, UDMA, HDDMA, PEGDMA, barium-boro-silicate glass (65 wt %)	20	Jeneric Pentron (Wallingford, CT, USA)
NX3	Composite resin cement	TEGDMA, bis-GMA, fluoro-aluminosilicate glass (67.5 wt %/47 vol %), activators, stabilizers, radiopaque agent	20	Kerr (Orange, CA, USA)
Variolink Esthetic DC	Composite resin cement	UMDA and further methacrylate monomers, ytterbium trifluoride, spheroid mixed oxide (67 wt %/38 vol %), initiators, stabilizers and pigments	10	Ivoclar Vivadent (Ellwangen, Germany)
Estecem	Adhesive resin cement	bis-GMA, TEGDMA, bis-MPEPP, silica-zirconia filler (74 wt %), camphorquinone	20	Tokuyama Dental (Taitou, Japan)
Multilink Automix	Adhesive resin cement	dimethacrylate and HEMA, barium glass and silica filler, ytterbiumtrifluoride (68 wt %), catalysts, stabilizers, pigments	10	Ivoclar Vivadent (Ellwangen, Germany)
Panavia 2.0	Adhesive resin cement	10-MDP, BPEDMA, hydrophobic aliphatic metahrylates, hydrophilic aliphatic metahrylate, silanated silica filler, silanated barium glass filler, sodium fluoride (70.8 wt %)	20	Kuraray (Osaka, Japan)
Breeze	Self-adhesive resin cement	bis-GMA, UDMA, TEGDMA, HEMA, 4-MET, silane treated barium glass, silica, BiOCl, Ca-Al-F-silicate, curing system	20	Jeneric Pentron (Wallingford, CT, USA)
Calibra Universal	Self-adhesive resin cement	UDMA, trimethylolpropane trimethacrylate TMPTMA, bis-EMA—Bisphenol A ethoxylate dimethacrylate, TEGDMA, HEMA, 3-(acryloyloxy)-2-hydroxypropyl methacrylate, urethane modified bis-GMA, PENTA, silanated barium glass, fumed silica (48 vol %)	10	Dentsply Sirona (York, PA, USA)
MaxCem Elite Chroma	Self-adhesive resin cement	HEMA, GDM, UDMA, 1,1,3,3-tetramethylbutyl hydroperoxide TEGDMA, fluoroaluminosilicate glass, GPDM, barium glass filler, fumed silica (69 wt %)	10	Kerr (Orange, CA, USA)
Panavia SA Cement Plus	Self-adhesive resin cement	bis-GMA, TEGDMA, HEMA, 10-MDP, hydrophobic aromatic dimethacrylate, hydrophobic aliphatic dimethacrylate, sodium fluoride, silanated barium glass filler, silanated colloidal silica (70 wt %/40 vol %)	10	Kuraray (Osaka, Japan)
RelyX U200	Self-adhesive resin cement	methacrylate monomers containing phosphoric acid groups, methacrylate monomers, silanated fillers (70 wt %/43 vol %), initiator components, stabilizers, rheological additives, alkaline(basic) initiator components, stabilizers, pigments	20	3M ESPE (St. Paul, MN, USA)
SmartCem 2	Self-adhesive resin cement	UDMA, urethane modified bis-GMA, TEGDMA, PENTA, dimethacrylate resins, barium boron fluoroaluminosilicate glass amorphous silica (69 wt %/46 vol %)	10	Dentsply Sirona (York, PA, USA)
SpeedCEM Plus	Self-adhesive resin cement	UDMA, TEGDMA, PEGDMA, methacrylated phosphoric acid ester, 1,10-decandiol dimethacrylate, copolymers, dibenzoyl peroxide, ytterbium trifluoride, barium glass, silicon dioxide (75 wt %/45 vol %)	20	Ivoclar Vivadent (Ellwangen, Germany)

bis-GMA—bisphenol A glycol dimethacrylate, UDMA—urethane dimethacrylate , TEGDMA—triethylene glycol dimethacrylate, GDM—glycerol 1,3-dimethacrylate, GPDM—glycerol phosphate dimethacryalte, bis-MPEPP—bisphenol A polyethoxy methacrylate, HEMA—hydroxyethyl methacrylate, PEGDMA—polyethylene glycol dimethacrylate, NPGDMA—neopentyldimethacrylate, 10-MDP—10-methacryloxydecyl dihydrogen phosphate, BPEDMA—bisphenol-A-polyethoxy dimethacrylate, PENTA—dipentaerythritol penttacrylate monophosphate, HDDMA—1,6-hexanediol dimethacrylate, 4-MET—4-methacryloyloxyethy trimellitate anhydride, MAC-10—11-methacryloyloxy-1,1-undecanedicarboxylic acid, TMPTMA—trimethylolpropane trimethacrylate.

**Table 2:** The curing time of bonding systems.

Bonding system	Manufacturer	Curing Time (s)	Bonding System Dedicated to
Bond-1 C&B Primer/Adhesive	Jeneric Pentrton (Wallingford, CT, USA)	10	Cement It, Breeze
Clearfil SE bond	Kuraray (Osaka, Japan)	10	Panavia 2.0, Panavia SA Cement Plus
Easy Bond	3M ESPE (St. Paul, MN, USA)	10	RelyX U200
Estelink	Tokuyam Dental (Taitou, Japan)	10	Estecem
Monobond Plus	Ivoclar Vivadent (Ellwangen, Germany)	10	Variolink Esthetic DC, Multilink Automix, SpeedCEM Plus
OptiBond XRT	Kerr (Orange, CA, USA)	10	NX3, MaxCem Elite Chroma
Prime&Bond Elect Universal	Dentsply Sirona (York, PA, USA)	10	SmartCem 2, Calibra Universal

**Table 3:** Stress state before and after 2016 h (84 days) of water immersion, contraction stress drop, absorbency, and solubility of tested materials.

Material	Stress State (MPa)		Contraction Stress Drop (%)	Sorption (µg/mm^3^)	Solubility (µg/mm^3^)
	0.5 h	2016 h			
Cement It	10.9 ± 2.2	−1.6 ± 0.4	115 *	27.8 ± 0.8	1.9 ± 0.4
NX3	6.3 ± 0.1	1.6 ± 0.1	79	23.8 ± 0.6	3.7 ± 1.2
Variolink Esthetic	10.9 ± 0.4	4.7 ± 0.1	57	22.4 ± 0.8	10.0 ± 2.0
Estecem	6.8 ± 0.9	1.6 ± 0.2	76	12.5 ± 2.2	4.6 ± 1.9
Multilink Automix	12.5 ± 0.4	2.1 ± 0.9	83	25.3 ± 1.5	2.2 ± 0.8
Panavia 2.0	5.3 ± 1.8	−4.8 ± 0.4	191 *	33,9 ± 1.7	11.1 ± 1.0
Breeze	7.8 ± 1.6	−6.3 ± 1.6	180 *	47.7 ± 3.1	3.1 ± 0.5
Calibra Universal	11.1 ± 0.7	0.0 ± 0.8	100	30.9 ± 1.5	5.0 ± 2.6
MaxCem Elite Chroma	10.4 ± 0.9	−6.3 ± 0.3	160 *	50.4 ± 1.3	8.5 ± 1.3
Panavia SA Plus	4.8 ± 0.4	−1.6 ± 0.2	133 *	26.4 ± 1.3	1.7 ± 0.4
RelyX U200	13.5 ± 0.8	2.6 ± 0.9	81	29.6 ± 1.3	0.4 ± 0.2
SmartCem 2	15.1 ± 0.9	1.6 ± 0.9	89	33.0 ± 0.9	4.9 ± 1.2
SpeedCEM Plus	11.9 ± 1.1	−1.6 ± 0.4	113 *	28.2 ± 0.5	2.5 ± 0.4

* represents materials with over-compensated polymerization stress due to water expansion.
